# Impact of Particle Size and Polydispersity Index on the Clinical Applications of Lipidic Nanocarrier Systems

**DOI:** 10.3390/pharmaceutics10020057

**Published:** 2018-05-18

**Authors:** M. Danaei, M. Dehghankhold, S. Ataei, F. Hasanzadeh Davarani, R. Javanmard, A. Dokhani, S. Khorasani, M. R. Mozafari

**Affiliations:** Australasian Nanoscience and Nanotechnology Initiative, 8054 Monash University LPO, Clayton, Victoria 3168, Australia; danagenepk@gmail.com (M.Da.); m_dehghan.kh@yahoo.com (M.De.); s.ataei@umsha.ac.ir (S.A.); Hasanzadeh.fatemeh1662@gmail.com (F.H.D.); r.javanmard@gmail.com (R.J.); info@anni.com.au (A.D.); dr.sepideh.khorasani@gmail.com (S.K.)

**Keywords:** drug delivery, encapsulation, lipidic nanovesicles, nanocarriers, particle size, toxicity

## Abstract

Lipid-based drug delivery systems, or lipidic carriers, are being extensively employed to enhance the bioavailability of poorly-soluble drugs. They have the ability to incorporate both lipophilic and hydrophilic molecules and protecting them against degradation in vitro and in vivo. There is a number of physical attributes of lipid-based nanocarriers that determine their safety, stability, efficacy, as well as their in vitro and in vivo behaviour. These include average particle size/diameter and the polydispersity index (PDI), which is an indication of their quality with respect to the size distribution. The suitability of nanocarrier formulations for a particular route of drug administration depends on their average diameter, PDI and size stability, among other parameters. Controlling and validating these parameters are of key importance for the effective clinical applications of nanocarrier formulations. This review highlights the significance of size and PDI in the successful design, formulation and development of nanosystems for pharmaceutical, nutraceutical and other applications. Liposomes, nanoliposomes, vesicular phospholipid gels, solid lipid nanoparticles, transfersomes and tocosomes are presented as frequently-used lipidic drug carriers. The advantages and limitations of a range of available analytical techniques used to characterize lipidic nanocarrier formulations are also covered.

## 1. Introduction

**Figure 1:**
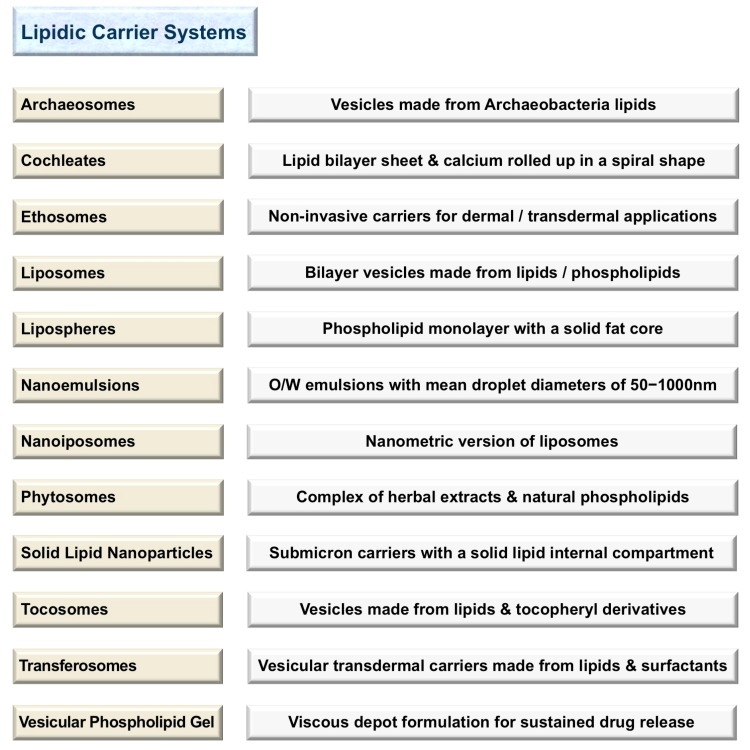
Main lipidic nanocarrier systems and a concise definition of each.

The number of products on the market manufactured using lipidic nanocarriers is increasing in parallel with increasing public awareness of the health benefits of such products. These products are mainly in the field of cosmetics, food/nutrition, nutraceuticals and pharmaceuticals. Lipid-based encapsulation systems are among the most promising technologies employed in drug delivery and sustained release of bioactive compounds. They include liposomes, nanoliposomes, archaeosomes, solid lipid nanoparticles (SLN), tocosomes and some other drug carrier systems. Figure 1 lists a number of currently available lipidic carrier systems and a brief description of each. One of the first and most applied drug delivery technologies is the liposome, which is also known as a bilayer lipid and/or a phospholipid vesicle. The word liposome has been adopted generally to refer to mesomorphic structures composed of lipid, phospholipid and water molecules. The main chemical components of liposomes are amphiphilic lipid/phospholipid molecules [1]. They improve the efficacy of pharmaceutical, nutraceutical and other bioactive compounds by entrapment and release of water-soluble, lipid-soluble and amphiphilic materials, as well as targeting the encapsulated compounds to particular cells or tissues [2]. Liposomes can be made on a small scale (e.g., for laboratory research) or industrial scales using natural ingredients such as soy or egg lecithin. However, it is also possible to include other molecules such as sterols (mainly cholesterol), polypeptides (e.g., antigens), polymers (such as poly-ethylene-glycol or chitosan), as well as antioxidants (e.g., α-tocopherol) in the structure of the lipid vesicles. These additives assist in targeting the lipidic vesicles (and their encapsulated molecules) where their effect is needed, or improving the stability and shelf life of the product. Nanoliposomes (lipidic nanovesicles or nanometric versions of liposomes), on the other hand, can be briefly defined as colloidal nanostructures composed of lipid/phospholipid bilayers [3]. In general, liposomes and nanoliposomes have the same physical, chemical and thermodynamic properties that are mainly determined by their ingredients and the media in which they are suspended. This is while the smaller the particle size, the larger the surface-to-volume ratio they will possess. Consequently, in comparison with liposomes, nanoliposomes provide more surface area and have more potential to increase solubility, enhance bioavailability, improve controlled release and enable accurate targeting of the encapsulated material. The manufacture of both liposomes and nanoliposomes requires the input of energy to a dispersion of lipid and phospholipid molecules in an aqueous medium [1,2,3,4,5]. Although lipid vesicles are initially prepared as a liquid suspension, they can be subsequently incorporated in a cream, lotion, aerosol, soft-gel, powder (upon freeze drying or spray drying for instance) or other formulations and dosage forms. Vesicular phospholipid gel (VPG) is an example of a highly concentrated phospholipid dispersion of semisolid consistency and vesicular morphology. VPG can be prepared by high-pressure homogenization using high concentrations of phospholipid molecules. Upon dilution with an aqueous solution, VPG constitutes a liquid liposome dispersion [6,7]. Solid lipid nanoparticles (SLN), on the other hand, are a recently-developed nanocarrier utilized as an alternative to the existing drug delivery technologies including polymeric nanocarriers, emulsions and liposomes. They are a new generation of submicron-sized lipidic carriers in which the liquid lipid (oil) has been substituted by a solid lipid, i.e., the lipid particle matrix being solid at room temperature, as well as body temperature [5]. Some of the solid lipids used in the preparation of SLN include triglycerides, emulsifying wax, cetyl alcohol, carnauba wax, beeswax, cholesterol and cholesterol butyrate. The underlying mechanism for the formation of the lipid vesicles is the hydrophilic–hydrophobic interactions and van der Waals forces between phospholipids and water molecules. There are a number of review articles describing the preparation methods of lipid-based vesicles, which readers are referred to for a broader coverage [1,2,5,8].

In addition to their application in the fields of encapsulation of bioactive compounds and drug delivery and targeting, lipid vesicles are being used as simplified models of cells and biological membranes. Their similarity to biomembranes makes them an ideal structure, not only for the study of existing biosystems, but also in the investigation of the emergence, functioning and evolution of original cells [9,10]. Applications of phospholipid vesicles in the area of food fortification are also rapidly growing. Food fortification is the process of adding micronutrients, including vitamins, minerals and essential fatty acids, to food products. These molecules and compounds may change the sensory qualities of food and adversely affect its smell, taste or colour. One of the main advantages of employing nanovesicles in the food industry is their ability to evade our sensory perception, enabling fortification of food and beverages with bioactive materials (such as omega fatty acids) without adversely affecting the sensory attributes of the original product [11,12,13]. If nanovesicles are kept below a size of around 80 nm in diameter (and not at very high concentrations, or particle refractive index not very different from that of the suspension medium), they hardly scatter any visible light and hence maintain transparency. Such invisible vesicles are very useful, for instance, for the fortification of clear beverages with hydrophobic molecules or those with undesirable odours or flavours [14]. The particle size distribution and polydispersity index (PDI) of lipid-based nanocarriers are highly important physical characteristics to be considered when creating food-grade or pharmaceutical-grade products. These attributes of the lipidic nanocarriers can affect the bulk properties, product performance, processability, stability and appearance of the end product. When formulating lipid-based bioactive carrier systems, a reliable and reproducible analysis of their mean diameter, heterogeneity and charge is important. Determination of the average diameter and determination of the size distribution of lipidic nanocarriers are fundamental quality control assays for such products [15]. In this review, the consideration of size and PDI parameters in the formulation and clinical utilization of lipidic nanocarriers are explained. The advantages and restrictions of a number of currently available analytical techniques used to characterize lipid-based nanocarrier formulations are also presented.

## 2. Impact of Particle Size

Different types of lipidic nanocarriers have been applied as drug delivery systems for diagnostic and targeted nanotherapy (employing active or passive targeting mechanisms) to achieve the maximum cellular uptake and therapeutic index [2,16,17]. Nanocarriers can be formulated and processed to differ in terms of composition, size, charge and lamellarity. Techniques such as extrusion, sonication, homogenization and/or freeze-thawing are being employed to control the size and size distribution of different drug carrier systems [1,2,3]. Continuous physicochemical improvements in the development of the lipid-based nanocarriers may have substantial implications in the cellular uptake and internalization, as well as the bioavailability of the encapsulated therapeutic compound. Particle size is a very critical attribute of lipidic nanocarriers, which affects stability, encapsulation efficiency, drug release profile, bio-distribution, mucoadhesion and cellular uptake [18].

**Figure 2:**
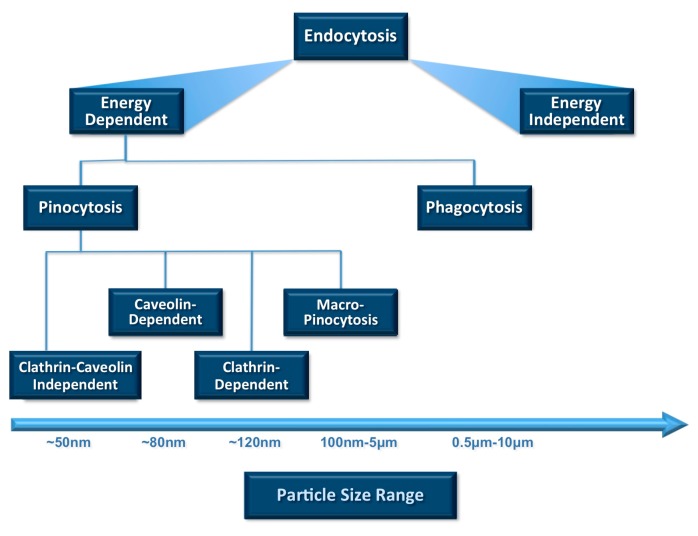
Relative sizes of particles and nanocarriers favourable for cellular uptake and ingestion through different endocytotic pathways. Vesicle size is one of the main parameters that determines clearance by the reticuloendothelial system (RES). The rate of uptake by the immune system cells increases by the increase in the size of the lipidic carriers.

Cellular uptake or internalization is one of the most important physicochemical criteria to be considered prior to in vivo applications. Uptake of small molecules and particles by any cell depends mainly on endocytosis among all other mechanisms (Figure 2). Endocytosis is the process of actively transporting materials into the cell by engulfing them with its phospholipid bilayer using energy in the form of ATP. The two main endocytosis mechanisms are reported to be pinocytosis and phagocytosis [19]. Cellular internalization by phagocytic cells such as macrophages, neutrophils and dendritic cells is mostly achieved by engulfing particles larger than 1 µm [20]. On the other hand, pinocytosis is another mechanism of endocytosis and involves taking extracellular fluids into the cells. Through pinocytosis, the cell can internalize fluids (including dissolved solutes) using a small amount of energy (in the form of ATP). It is mainly associated with particle uptake by the cells via different pathways such as macro-pinocytosis, clathrin-mediated, caveolin-dependent and caveolin-independent pinocytosis, as depicted in Figure 2. The particle size and PDI of nanocarrier systems are the main physicochemical attributes that influence the endocytosis-dependent cellular uptake.

### 2.1. Impact of Particle Size on Systemic Drug Delivery

It is well documented that the size of drug delivery systems influences pharmacokinetics, tissue distribution and clearance. Certain physiological processes such as hepatic uptake and accumulation, tissue diffusion, tissue extravasation and kidney excretion significantly depend on particle size. Only nanocarriers, including SLN, of a certain size (≤150 nm) are able to enter or exit fenestrated capillaries in the tumour microenvironment or liver endothelium [21,22]. Nanocarriers circulating in normal blood vessels do not easily leave the capillaries that perfuse tissues such as the kidney, lung and heart if they have a diameter range of 100–150 nm [18,22]. However, smaller particles in the size range of 20–100 nm may distribute to bone marrow, spleen and liver sinusoids and may leave the bloodstream via the leaky capillaries of these organs to some extent. It is known that lung alveoli may trap particles of several micrometres in diameter, and the pore size of the pulmonary capillary barrier is estimated to be approximately 35 nm [23]. This pore size is two- to three-times lower than that of the pores within the endothelial lining of kidney capillaries. Glomerulus in the kidneys and islet tissues in the pancreas have smaller pores with diameters around 10–15 nm [24]. Particles with diameters less than 10 nm experience renal filtration via the wall of the glomerular capillary and are not reabsorbed. These tissue and capillary pore size ranges are the reason why most nanocarriers of 50–200 nm in size in their intact form are not able to escape from continuous blood capillaries. Nevertheless, when extravasated from blood vessels (typically via discontinuous capillaries in the bone marrow, liver, spleen and to some extent in the lungs), liposomes and lipidic nanocarriers larger than 100–150 nm can be taken up by phagocytes or remain in these tissues for an extended time [25]. The majority of these phagocytes accumulate in the liver and spleen for subsequent elimination. Once in a tissue, lipidic nanocarriers could be retained because of the capillary pore size or dimensions of the interstitial space of the tissue [26,27].

### 2.2. Impact of Particle Size on Pulmonary Drug Delivery

Drug administration to human lungs is advantageous for local treatments of diseases such as cystic fibrosis, lung cancer, asthma or other related respiratory distress syndromes. This route can also be applied for systemic delivery of bioactive materials such as peptides and nucleic acids, which are unstable in the gastrointestinal tract, for instance. The advantage of topical drug administration to the lung is the potential of delivering an adequate drug dose to the target site with reduced undesirable extrapulmonary side effects. There are many distinct advantages of lipidic carriers, which make them particularly attractive for drug administration to the lung. These favourable attributes include biocompatibility, ideal specific gravity, targetability and the possibility of producing them in diverse size ranges [28]. The attractiveness of using phospholipid-based carriers (e.g., liposomes and nanoliposomes) as a pulmonary drug delivery system also stems from the fact that phospholipids are naturally-occurring components of lung surfactant and, therefore, should not pose a toxicological risk to this organ [28,29].

When drug carrier systems are intended for inhalation, their size distribution is of primary consideration, since it influences the in vivo fate of the carrier system and the encapsulated therapeutic molecules. It is known that lung deposition of an aerosol depends on its mean aerodynamic particle size, which can also impact the clinical effectiveness of the therapeutic agent [29,30,31,32]. It has been postulated that aerosol particle size characteristics can play an important role in avoiding the physiological barriers of the lung, as well as targeting therapeutic compounds to the appropriate pulmonary region [29]. However, it is difficult to predict the actual site of drug deposition, due to the fact that airway calibre and anatomy differ among people. In general, aerosols with a mass median aerodynamic diameter (MMAD) of 5–10 µm are mainly deposited in the large conducting airways and oropharyngeal region [30]. Particles with a 1–5 µm MMAD range are deposited in the small pulmonary airways and alveoli, whereas more than 50% of the particles with 3 µm MMAD are deposited in the alveolar region. In the case of employing the pulmonary route for systemic drug delivery, aerosols with a small average particle size are required to ensure peripheral penetration of the drug [31,32]. Particles smaller than 3 µm have an approximately 80% probability of reaching the lower airways, while around 50–60% of these particles will be deposited in the alveoli [20,29]. On the other hand, nanosized carrier systems have recently gained increasing attention for pulmonary drug delivery. This is due to their advantages for targeted deposition, bioadhesion, sustained release and reduced dosing frequency to improve patient convenience [33,34]. While the most effective particle size for the treatment of systemic diseases has not been determined yet, particles smaller than 150 nm are reported to have delayed lung clearance, increased protein interactions and more transepithelial transport compared to larger particles [33,35]. 

### 2.3. Impact of Particle Size on Drug Delivery to Tumours

Particle size is one of the main parameters employed to passively target therapeutic agents to tumours [36]. The tumour vasculature is very different from that of the normal tissues. They are larger in size, more heterogeneous in distribution, have high vascular density and are more permeable and leaky [37]. Consequently, there will be accumulation of vascular mediators at the tumour sites along with the impaired lymphatic drainage of macromolecules. The leaky vasculature of tumours allows accumulation of high molecular weight therapeutics in the tumours. This phenomena is known as the enhanced permeability and retention (EPR) effect, which eventually enables the circulating nanocarriers smaller than around 150 nm to extravasate from circulation through the tumour vasculature and increase the concentration of the chemotherapeutic agents within the tumour [36,37,38]. However, some literature mentions a size of below 200 nm for passive targeting tumour tissues via EPR [39,40].

It has been reported that decreasing the nanoliposome size to 50 nm in diameter or below greatly reduced mononuclear phagocyte system (MPS)-mediated clearance in mice models and achieved a plasma half-life comparable to that achieved by long-circulating (PEGylated) vesicles with 100–150 nm diameters [41,42]. MPS uptake can be prevented or reduced by saturating the blood circulation with high doses of nanoliposomes containing the encapsulated active compound or by predosing with large quantities of control (empty) nanoliposomes to inhibit phagocytic activity. These strategies may not be effective for clinical applications due to the adverse effects resulting from the destruction of phagocytic functions of the MPS (a natural mechanism to protect the body from pathogenic invasions). Consequently, to avoid MPS uptake and to prolong blood circulation time, most therapeutic nanoliposomes are designed to possess 50–100 nm diameters. For instance, DaunoXome (a nanoliposomal anticancer formulation) consists of 50–80 nm diameter particles intended to reduce MPS uptake [43,44]. Serum protein binding and associated complement-dependent activation are reported to be dependent on nanoliposome size [45]. These two mechanisms together increase the rate of particle clearance in vivo [46]. Nanoliposomes with diameters less than 50–80 nm are subject to significantly lower MPS-dependent clearance in humans. Once PEGylated, vesicles with diameters less than 100–150 nm exhibit reduced plasma protein binding, as well as decreased hepatic and MPS uptake. The presence of PEG polymer coating on the surface of the lipidic carriers has been shown to reduce their uptake by phagocytic cells. As a result, these long-circulating carriers (also known as stealth vesicles) attain longer blood circulation times [45,46]. 

For the treatment of lung cancer, inhalable nanocarriers have gained more attention in recent years. This is due to their ability to highly associate with therapeutic agents and sustain their release. Moreover, they can be targeted to cancer tissues in the lungs and have the ability to be efficiently transferred into aerosols and highly endure nebulization forces [47]. Lipidic nanocarriers can avoid mucociliary clearance and lung phagocytic mechanisms, thus prolonging the residence of the therapeutic agent within the pulmonary system [48]. The particle size of the aerosol plays a crucial role in specific targeting to different lung regions based on the position of diseased cells within the lung. Larger aerosol particles with diameters of 5–10 μm are principally deposited in oropharynx and large airways, while smaller particles with diameters of 1–5 μm are located in the small airways and alveoli [49]. Nanocarriers in the size range of 100–150 nm display 8–9-times more internalization into lung tumour cells compared to microparticles with a size range of 3–5 μm [50]. Consequently, precise tailoring of aerosol particle size is required to achieve deep lung deposition and the best internalization into the tumour cells. 

### 2.4. Impact of Particle Size on Transdermal Drug Delivery 

Transdermal delivery of therapeutic agents involves the application of the formulation to the intact skin and delivery of the drug at a controlled rate locally or to the systemic circulation. As a convenient route of drug administration, transdermal drug delivery has made an important contribution to medical practice. However, it has yet to achieve its full potential as an alternative to oral delivery and hypodermic injections [51]. The mechanisms involved in transdermal drug delivery applications depend on the formulation of nanocarriers; in particular, factors such as chemical composition, surface charge, number of lamella and particle size must be carefully considered. The first studies on exploring the potential use of lipid vesicles in topical applications for the skin were reported in the 1980s [52,53,54]. Phospholipid vesicles have proven to be useful in the treatment of skin diseases such as psoriasis and skin cancer [55]. Through the utilization of transdermal dosage forms, bioactive compounds can be targeted to the site of the infection or disease and side effects can be kept to a minimum by the prevention of systemic absorption of the drug [56]. The particle size of lipidic vesicles has been shown to have a significant influence on bioactive delivery into the skin [57,58]. Generally, vesicles with a diameter of 600 nm or above are not able to deliver the encapsulated material into deeper layers of the skin. These vesicles are inclined to stay in or on the stratum corneum and may form a lipid layer on the skin after drying [57,58,59].

Nanovesicles with a diameter of 300 nm or below are able to deliver their contents to some extent into the deeper skin layers. However, nanovesicles with a diameter of 70 nm or below have shown maximum deposition of contents in both viable dermal and epidermal layers [57,59]. Nanoparticles below 6–7 nm in size can be absorbed through the lipidic transepidermal routes, while those with a particle size of below 36 nm can be absorbed through the aqueous pores. Particles in the size range of 10–210 nm, however, may preferentially penetrate through the transfollicular route [60,61]. There are specialized lipid-based encapsulation systems for topical and transdermal drug delivery based on skin penetration enhancement mechanisms and/or certain molecules referred to as “edge activators” [61,62]. They include transfersomes [63], ethosomes [64], solid lipid nanoparticles [65] and the more recently introduced tocosomes [66], a brief definition of which is given in Figure 1 (for a detailed description of these specialized drug delivery systems, see [61,62,63,64,65,66,67]). 

### 2.5. Impact of Particle Size on Drug Delivery to Brain

The impermeable characteristic of the blood brain barrier (BBB) has been considered to be the main reason for the failure to achieve therapeutic drug concentrations in the brain tissue. There are fundamental differences between brain capillaries and peripheral capillaries. While peripheral capillaries are fenestrated with gaps up to 50 nm wide, brain capillary endothelial cells are closely connected to each other by tight intercellular junctions and zonulae occludentes [68,69]. The BBB prevents many therapeutic agents, including peptides and medicinal macromolecules, from entering the brain and the rest of the central nervous system (CNS). Consequently, many researchers have tried to overcome the BBB for therapeutic purposes in several different CNS disorders [70,71]. However, these trials have been hampered by limited information on the molecular basis of BBB. A number of therapeutic compounds has proven to be ineffective in the treatment of cerebral diseases due to difficulties to deliver and sustain these drugs within the brain efficiently. As a result, any method that can enhance drug delivery to the brain is of great interest.

In a study aimed at overcoming BBB and targeting brain tumour, Zong et al. [72] prepared doxorubicin-loaded liposomes containing two peptides (TAT and T7) as targeting moieties. The formulation exhibited an improvement in the therapeutic efficacy in the treatment of glioma in animal models as compared to vesicles containing a single targeting moiety and free doxorubicin [72]. Recently, Zhang et al. [73] reported the formulation of PEGylated nanoliposomes within the size range of ca. 93–96 nm, encapsulating two anticancer agents (vincristine and doxorubicin), for the treatment of brain glioma. The nanoliposomes were composed of distearoyl- phosphoethanolamine (DSPE) conjugated with polyethylene glycol (PEG) and two targeting ligands (i.e., T7 and DA7R peptides). The dual targeting strategy resulted in higher therapeutic efficacy as a result of improved drug delivery to the brain of glioma-bearing mice [73]. Another study similarly reported successful drug targeting to brain tumour and overcoming BBB in mice using nanovesicles with a ca. 100-nm average diameter [74]. The approximate particle size range for drug deposition in the brain and some other body organs depending on the dosage form and route of administration are presented in Table 1.

## 3. Polydispersity Index

The safety and efficacy of therapeutic compounds are limited by inadequate drug delivery to the target tissue or undesired side effects such as severe toxicities in healthy tissues and organs. Both of these concerns can be addressed by encapsulating the drug inside lipidic nanocarriers with defined and predictable characteristics, which provide maximum bioavailability and minimal side effects. The tendency of lipidic nanocarriers to accumulate in the target tissue depends on their physicochemical characteristics including particle size distribution. Successful formulation of safe, stable and efficient nanocarriers, therefore, requires the preparation of homogenous (monodisperse) populations of nanocarriers of a certain size. However, it is difficult to control the particle size distribution without considering the composition of the nanocarriers and the nature of the solvents and co-solvents used during their preparation [8,75,76]. Following preparation, nanocarriers must be characterized to assure their suitability for in vitro and in vivo applications. With respect to particle size distribution characterization, a parameter used to define the size range of the lipidic nanocarrier systems is called the “polydispersity index” (PDI). The term “polydispersity” (or “dispersity” as recommended by IUPAC) is used to describe the degree of non-uniformity of a size distribution of particles [77,78]. Also known as the heterogeneity index, PDI is a number calculated from a two-parameter fit to the correlation data (the cumulants analysis). This index is dimensionless and scaled such that values smaller than 0.05 are mainly seen with highly monodisperse standards. PDI values bigger than 0.7 indicate that the sample has a very broad particle size distribution and is probably not suitable to be analysed by the dynamic light scattering (DLS) technique (explained more in the next section). Different size distribution algorithms work with data that fall between these two extreme values of PDI (i.e., 0.05–0.7). The calculations used for the determination of size and PDI parameters are defined in the ISO standard documents 13321:1996 E and ISO 22412:2008 [79].

In the field of polymer science, PDI is employed to measure the breadth of the molecular weight distribution (MWD) of the polymer. PDI can be defined as Mw/Mn, where Mw is the weight average and Mn is the number average molecular weight [80,81]. In the fields of molecular science (using chromatography techniques), nanotechnology and nanoparticle research (using light scattering), there are in principle two different aspects of polydispersity, depending on the property of interest. In size exclusion chromatography and gel permeation chromatography, the property of interest is the molecular weight of the sample. The distribution obtained from these techniques is typically a molecular weight distribution describing how much material there is in each of the different molecular weight “segments”. When employing the DLS technique, however, the property of interest is the size distribution of molecules, particles or nanovesicles. The distribution describes how many vesicles there are in each of the various size “segments” [77,78].

**Figure 3:**
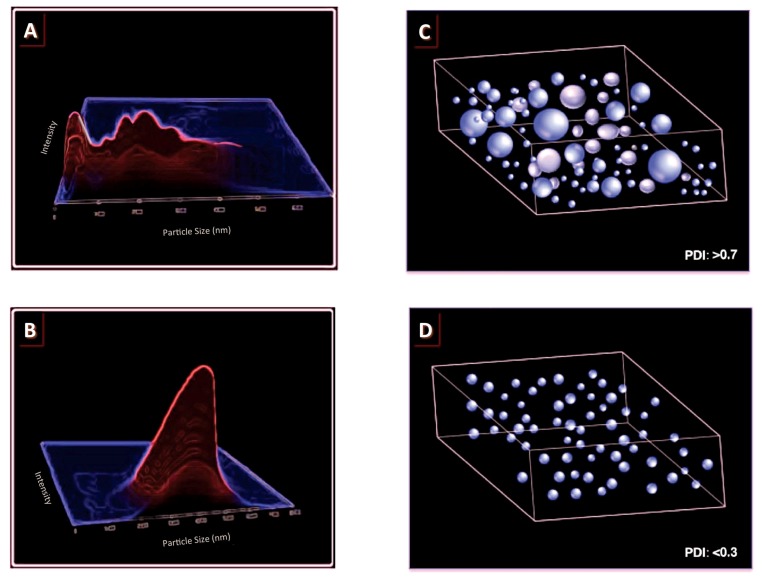
(**A**,**B**) schematic representation of typical particle size graphs indicating a polydisperse sample (composed of heterogeneous population of particles) (**A**); and a monodisperse sample (containing homogenous population of particles) (**B**); (**C**,**D**) representation of the particle size distribution of a sample containing a polydisperse population of particles (with a high PDI value) (**C**); and a sample containing a monodisperse population of particles (with a low PDI value) (**D**).

PDI is basically a representation of the distribution of size populations within a given sample. The numerical value of PDI ranges from 0.0 (for a perfectly uniform sample with respect to the particle size) to 1.0 (for a highly polydisperse sample with multiple particle size populations). Values of 0.2 and below are most commonly deemed acceptable in practice for polymer-based nanoparticle materials [82]. In drug delivery applications using lipid-based carriers, such as liposome and nanoliposome formulations, a PDI of 0.3 and below is considered to be acceptable and indicates a homogenous population of phospholipid vesicles [83,84,85]. Although the last edition of the FDA’s “Guidance for Industry” concerning liposome drug products [86] emphasizes the importance of size and size distribution as “critical quality attributes (CQAs)”, as well as essential components of stability studies of these products, it does not mention the criteria for an acceptable PDI. More specific standards and guidelines for the acceptability of product PDI range for different applications (e.g., food, cosmetic, pharmaceutical, etc.) and different routes of bioactive administration need to be set by the regulatory authorities. Figure 3 schematically represents the relationship between the particle size distribution and PDI values.

## 4. Methods of Analysis

The benefits of the delivery of therapeutics by nano-sized encapsulation systems is an area of much debate, and a better understanding of the possible mechanisms and opportunities to enhance tissue selective uptake could provide clues to obtaining more clinically significant delivery outcomes. Advances in approaches to the preparation of lipidic nanocarriers have provided new opportunities to fine-tune their particle size distributions and PDI. Various techniques of determining the size of the lipidic nanocarriers include microscopy (e.g., optical microscopy, negative stain electron microscopy, cryo-transmission electron microscopy, scanning electron microscopy, confocal microscopy and scanning probe microscopy), diffraction and scattering techniques (laser light scattering and photon correlation spectroscopy) and hydrodynamic techniques (field flow fractionation, gel permeation chromatography, ultracentrifugation and centrifugal sedimentation). The other available techniques for size analysis of nanoliposomal formulations are fluorescence microscopy, coulter counter, flow cytometry and the optical density method [15,77,87,88,89,90,91,92]. There are many reports on the usefulness of these techniques in providing complementary information regarding liposomes and nanoliposomes, as well as characterization of other lipid-based structures [90,91,92,93]. Ideally, methods of characterization of nanocarriers have to be meaningful, reproducible and rapid. Microscopic methods are widely used in order to establish the morphology, lamellarity, surface characteristics, size and stability of nanocarriers. With respect to a statistically meaningful analysis of the size distribution of nanocarrier formulations, methods such as light scattering, which measures the size distribution of a large number of particles in an aqueous sample instantaneously, are more applicable than microscopic techniques [3,91]. DLS is a noninvasive technique, which offers good statistics with respect to the in situ measurements of the size and PDI of nanocarriers, and also, it allows particle sizing down to 1 nm in diameter [15,77,91]. However, it does not provide information regarding the morphology and shape of the lipidic system (e.g., oval, spherical, cylindrical), and it assumes any aggregation of several vesicles as one single particle. Microscopic techniques, on the other hand, give a more detailed view of the morphology of nanostructures. They make direct observation of the sample possible and as such provide information about the shape of the nanocarriers, as well as the presence/absence of any aggregation and/or fusion. Freeze fracture electron microscopy, for instance, can also make it possible to visualize the number of vesicle bilayers (lamellarity) and internal compartments. Some of the main disadvantages of the microscopic techniques, in general, are that the number of particles that can be analysed in the sample is limited and the sample preparation can be tedious. The general approach for the characterization of nanocarrier formulations should hence be to employ as many of the above-mentioned techniques as possible.

Another type of modern microscopic technique, widely used to analyse nanocarriers with high resolution, is scanning probe microscopy (SPM). SPM is a technique for imaging surfaces at the nanometre scale by rastering a fine probe (also known as a tip) across the surface and measuring the repulsive/attractive interactions between the tip and the surface. SPM is a general term comprising a wide variety of techniques based on different interactions between the tip and the surface. These techniques, defined by the type of interaction being measured, include atomic force microscopy (AFM), scanning tunnelling microscopy (STM), magnetic force microscopy (MFM), electrostatic force microscopy (EFM) and Kelvin probe force microscopy (KPFM). Unlike most of the other microscopic techniques, scanning probe microscopes do not require extensive sample preparation procedures [90,94,95]. SPM techniques can be used to study nanostructures in air or solution at ambient conditions with simple sample preparation procedures. The majority of other microscopic techniques involve sample manipulation procedures such as staining, labelling, fixation or vacuum, which may cause some alterations in the structure and/or size of the lipidic nanocarriers [88,96].

Small-angle X-ray scattering (SAXS) is another technique by which nanoscale density differences in a sample can be quantified. This method can determine size distributions and resolve the size and shape of (monodisperse) nanocarrier samples. SAXS provides complementary information about folding and unfolding of macromolecules in addition to extended conformations, flexibly linked domains, aggregation/fusion of particles, shape and assembly state of samples in solution, at the resolution range of approximately 10 A–50 A, without the size limitations encountered in the electron microscopy methods and NMR [97].

There are some other analytical techniques, which make the assessment of size and PDI of single individual nanocarriers, possible. One of these methods is “scanning ion occlusion sensing” (SIOS), which is a nanopore-based technology that can be used for single-particle analysis [98]. SIOS analyses lipid-based nanocarriers in the size range of 60 nm to a few micrometres [99]. The operation mechanism of SIOS is based on the conventional Coulter counter, where individual particles are measured as they traverse a nanopore. When an individual particle or vesicle passes through the tuneable nanopore, a current reduction occurs due to an increase in the electrical resistance. The extent of current reduction and the frequency of the pulses are related to the particle size and concentration of the nanocarrier sample, respectively. Particles are driven either by electrophoresis and electroosmosis or by pressure generated from a pressure module [100,101]. SIOS is a useful method to analyse multiple parameters of nanocarriers on a particle-by-particle basis. This technique has proven to possess higher resolution in comparison with techniques such as dynamic light scattering. Furthermore, SIOS was successfully used to measure changes in the size and surface charge of phospholipid vesicles as a result of incubation in plasma [98,100]. There are still some limitations and issues that need to be improved with SIOS analysis. For instance, it is problematic to choose a suitable elastic pore for polydisperse samples to avoid detecting several particles at the same time. Moreover, it is still difficult to detect only one particle at the time, and the data acquired with different nanopore sizes cannot be compared in parallel [101].

An established method for the assessment of a single nanocarrier diameter and size distribution is flow cytometry (FCM). This technology is widely employed in analysing and sorting cells, bacteria, and other cell-sized particles. FCM has been applied in the analysis of multilamellar and large unilamellar vesicles (MLV and LUV) [102]. It employs light scattering to measure particles and vesicles in a continuous flow system. Samples have to be fluorescently labelled in order to be distinguished from the impurities and noise signal. Consequently, the scattered light at a 10° angle, or side scattered light at a 90° angle, or the fluorescence of the sample is measured. FCM is a very quick, reliable, robust and reproducible method. However, when employing light scattering detection, its operation can be disturbed by noisy signals from buffers, optics or electronics [98,101,102].

Nanoparticle tracking analysis (NTA) is another technology that is able to track and measure a single nanocarrier moving under Brownian motion [103]. NTA is a high-resolution method and effective at measuring the size, size distribution and concentration of liposome and nanoliposome samples. It can be employed to measure the size of the vesicles and particles within a size range of 30–1000 nm [104]. For NTA analysis, samples are injected into the special cell and then illuminated by laser light (635 nm) that passes through a liquid layer on the optical surface [104,105]. Refraction occurs, and the region in which the lipid-based nanocarriers are present is illuminated and visualized under the microscope. A charge-coupled camera records a video (30 frames per second) in which the motion of nanocarriers (Brownian motion) could be observed. Computer software identifies and tracks the centre of each object throughout the length of the video and relates it to the size of the vesicles. The hydrodynamic size and size distribution of the nanocarriers can be calculated by the Stokes–Einstein equation using a particle diffusion coefficient. This method enables measurement of the size of both monodisperse and polydisperse samples. Furthermore, it is able to measure the surface charge of the lipid-based carriers and detect their fluorescence signals. The drawbacks of NTA method, however, include its requirement for complex optimization by a skilled operator and the difficulty to identify an appropriate concentration of the sample. Furthermore, characterization employing the NTA technique can be hindered by the refractive index of the sample [106,107].

## 5. Conclusions

The use of lipid-based nanocarriers in medicinal and non-medicinal formulations has been reported mainly at laboratory scales, and many resulting nanomedicines are in the transition phase towards clinical applications. Trends of the global market have been indicating strong growth of the nanotherapy sector in the next few years. The translation of nanomedicines to the clinical phase and subsequent commercialization requires research, development and characterization of new formulations to ensure the quality, safety and efficacy of such products. Size variations of nanocarrier systems over time must be seriously considered when formulating encapsulated therapeutic agents. Nanocarrier formulations with a constant and narrow size distribution are necessary to achieve optimum clinical outcomes. Moreover, particle size and size distribution are important factors for evaluating the stability of a colloidal dosage form upon storage. The size stability issue is more crucial for nanosystems than for microscale drug delivery systems. This is due to the fact that nanosystems have a large specific surface area compared to microsystems. A number of available techniques for the evaluation of the size and PDI of nanocarriers were described in this entry along with their advantages and limitations. The regulatory agencies will benefit from information on the performance of novel or modified particle characterization techniques, which can be applied to the research and development of the current and next generation of nanotherapeutic agents.

## Figures and Tables

**Table 1:** Approximate particle size range for drug deposition in various body organs via different dosage forms and routes of administration.

Route of Administration/Dosage Form	Particle Size Range
Lymphatic (RES) *	10–50 nm
Long-circulating carriers (brain, tumour)	50–200 nm
Transdermal	10–600 nm
Intravenous/intramuscular	200–2000 nm
Ocular	100–3000 nm
Aerosol	1–10 µm
Nasal	8–20 µm

* Reticuloendothelial system.
